# Trajectories in suicide attempt method lethality over a five-year period: Associations with suicide attempt repetition, all-cause, and suicide mortality

**DOI:** 10.1371/journal.pone.0245780

**Published:** 2021-01-22

**Authors:** Katrina Witt, Jane Pirkis, Debbie Scott, Karen Smith, Dan Lubman

**Affiliations:** 1 Turning Point and Eastern Health Clinical School, Monash University, Melbourne, Australia; 2 Monash Addiction Research Centre, Monash University, Melbourne, Australia; 3 School of Population and Global Health, The University of Melbourne, Melbourne, Australia; 4 Centre for Research and Evaluation, Ambulance Victoria, Melbourne, Australia; 5 Department of Epidemiology and Preventive Medicine, Monash University, Melbourne, Australia; 6 Department of Community Emergency Health and Paramedic Practice, Monash University, Melbourne, Australia; University of New South Wales, AUSTRALIA

## Abstract

It is not known if there are discernible patterns in method lethality over successive episodes of self–harm and, if so, how these may be differentially associated with risks of self–harm repetition and suicide. Latent trajectory modelling estimated variation in patterns of suicide attempt lethality in 1,719 individuals attended by ambulance services on at least three occasions between 2012 and 2016. Cox regression modelling investigated hazards of suicide attempt repetition, all–cause, and suicide mortality as a function of these patterns. Two distinct trajectories provided optimal fit (BIC: –39,464.92). The first (Low/Moderate to Low/Moderate Lethality group; 92.5%) consisted of those consistently using methods associated with low to moderate potential lethality throughout the observation period. The second (High to Low/Moderate Lethality group; 7.5%) consisted of those who initially used methods with higher potential lethality but who switched to methods characterised by lower lethality. There were no significant differences between groups in the hazards of reattempting suicide (Hazard Ratio [HR] = 1.41, 95% CI 0.76 to 2.59) or all–cause mortality (HR = 1.21, 95% CI 0.63 to 2.32). However, those assigned to the High to Low/Moderate Lethality trajectory group may be at greater risk of suicide (Sub–Hazard Ratio [SHR] = 2.82, 95% CI 1.16 to 6.86). There may be discernible sub–groups of patients with important differences in clinical treatment needs and suicide risk profiles. These differences should be considered when undertaking psychosocial risk/needs assessments with those presenting to clinical services following self-harm.

## Introduction

Suicide is the leading cause of death for Australians between 18 and 44 years of age [1], and the leading cause of injury–related death for Americans between these ages [2]. For every suicide death, there are an additional six [3] to 40 [4] suicide attempts resulting in presentation to the emergency department (ED). A history of attempted suicide is one of the strongest risk indicators for both future suicide attempts and death [5].

The risks of both suicide death and subsequent suicide attempts are particularly elevated for those who make multiple suicide attempts, defined as a history of three or more attempts [6]. Those who make multiple attempts are more likely to report greater psychiatric morbidity, particularly symptoms related to anxiety and substance use disorders [7, 8], and are more likely to receive higher suicide risk scores, indicating the use of methods associated with greater potential lethality [8]. Despite this, those who make multiple attempts are more likely to leave the ED without receiving a full psychosocial risk/needs assessment despite clinical practice recommendations to the contrary [9].

The risks of suicide and suicide attempt repetition may also be influenced by the method used at the index (or first recorded) episode [10, 11]. However, previous studies have produced mixed findings regarding whether there is a discernible pattern in evolution of methods over successive episodes and, if so, how these may influence repetition and/or suicide risk [12]. Previous studies have either tended to focus on the association between the method used at the index hospital presentation, or at the episode immediately prior to a repeat episode and/or suicide [12]. Additionally, these studies have tended to group methods into self–injury and/or self–poisoning with little consideration as to the diverse range of methods included within these broad categories, likely differences in clinical treatment needs between individuals using these different methods, and likely differences in potential lethality between methods [12].

We used a latent variable classification method to investigate whether there are discernible trajectories of suicide attempt lethality for those making multiple suicide attempts. We also investigated whether these groups differed in terms of their demographic and/or clinical characteristics. Finally, we conducted a data linkage exercise to understand whether there were differences between groups in the risks of suicide attempt repetition, all–cause mortality, and suicide mortality.

## Materials and methods

This study examined a retrospective cohort of all ambulance attendances for attempted suicide (which we defined as any intentional injury with a non-fatal outcome for which there is either explicit or implicit evidence of suicidal intent) in Victoria, Australia over a five-year period (1 January 2012 and 31 December 2016). The data collection process has been described in detail elsewhere [13–15]. Extracts of electronic records for suicidal behaviour-related attendances were received as direct data imports from Ambulance Victoria. Each record was subsequently coded by trained research assistants to identify information on patient demographics, self-harm method, alcohol and other drug (AOD) co–involvement, self–reported psychiatric morbidity, and acute psychiatric symptomatology.

To estimate the potential lethality of each method, we calculated case fatality ratios (CFRs) using annualised counts of suicide deaths per annum over this period [16], alongside data on the number of hospitalised cases [17]. At the time of writing, 2014–15 (i.e., the mid–point for this study) was the latest year for which data on hospitalised cases of attempted suicide by primary mechanism of injury were available. Five categories of methods were used, defined by the Australian modification of the International Classification of Disease version 10 (ICD–10–AM) codes as: intentional drug overdose (X60–X64), poisoning by other substances (X65–X69), hanging, strangulation, and suffocation (X70), use of firearms or explosives (X72– X74), self–cutting, laceration, and stabbing (X78), drowning and submersion (X71), falls from a height (X80), and other methods (X75–X77, X79, X81–X84). Where a single attempt involved multiple methods, we used the CFR for the method associated with the highest potential lethality.

### Statistical analysis

Rates of suicide attempt-related attendances per 100,000 person–years were calculated using mid–year estimated resident population data for 2012 to 2016 [18]. Negative binomial regression models were used to investigate changes in rates of suicide attempt–related attendances per 100,000 person–years for all persons, and for males and females separately. Data from a period spanning three months in 2014 (October, November, and December) were unavailable due to a lapse in electronic data collection as a result of paramedic industrial action [19]. Data for these three months were therefore imputed using methods previously applied to Ambulance Victoria data to facilitate time series analysis [19]. Imputed data were not used in any subsequent analysis.

Given that latent trajectories identified from fewer than three independent observations are likely to be unstable [20], all subsequent analyses were limited to those with three or more attempts, consistent with international definitions of multiple suicide attempt repetition [6]. Regression models and Cramér’s V were used to indicate whether these groups differed on a number of key demographic and clinical factors. Cramér’s V can take any value between zero and one, with values >0.00≤0.10 indicating negligible associations, values >0.10≤0.30 indicating small associations, values >0.30≤0.50 indicating medium associations, and values >0.50 indicating large associations [21].

Next, latent trajectory modelling was used to characterise trajectories of suicide attempt method lethality [22]. A two–stage process was used to determine both the optimal number of trajectories to retain and to define the shape of these [22]. Model fit was determined by the sample size adjusted Bayesian Information Criterion (BIC) alongside a number of other fit statistics as recommended [22]. Regression models were used to investigate the extent to which a series of both time invariant and time varying demographic and clinical covariates were associated with method lethality for each trajectory group. Specifically, sex was modelled as a time invariant covariate (with male sex indicating exposure), whilst self–reported lifetime diagnoses of a number of psychiatric disorders, acute alcohol intoxication, and illicit drug co–involvement were modelled as time varying covariates [22].

Cox regression modelling was used to investigate the hazards of suicide attempt repetition, all–cause mortality, and suicide death by trajectory group. Data on subsequent ambulance attendances for attempted suicide were obtained from Ambulance Victoria, whilst information on all–cause and suicide mortality were obtained by linking ambulance data to the Victorian Deaths Index (VDI). The Centre for Victorian Data Linkage (CVDL) undertook probabilistic data linkage to ascertain dates and cause of deaths until 31 December, 2017. Death was treated as a censoring event for all analyses. Cohort members were followed until the date of their death or the end of the follow–up period (31 December, 2017), whichever came first.

This study was approved by the Eastern Health Human Research Ethics Committee and all analyses were undertaken using Stata for Windows, version 13.

## Results

**Fig 1:**
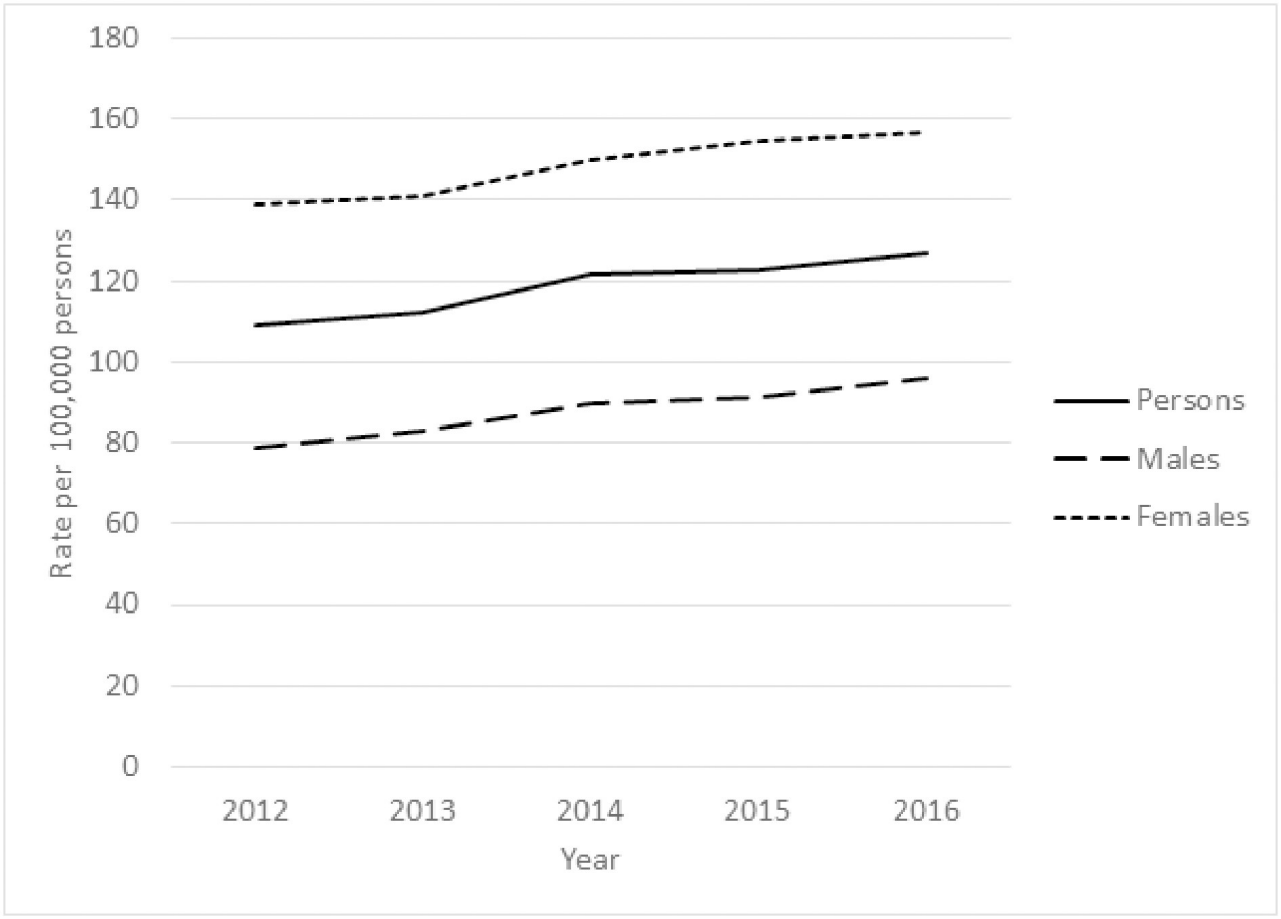
Rates of suicide attempt-related ambulance attendances per 100,000 estimated resident population in Victoria, 2012–2016.

Between 1 January 2012 and 31 December 2016, Victorian ambulance paramedics responded to a total of 33,448 suicide attempt-related attendances. There was evidence of a 22% increase in rates of suicide attempt–related attendances per 100,000 persons over this period (Incidence Rate Ratio [IRR] 1.04, 95% CI 1.04 to 1.04), as well as for males (28% increase; IRR 1.05, 95% CI 1.04 to 1.05) and females (16% increase; IRR 1.03, 95% CI 1.03 to 1.04) separately (Fig 1).

**Fig 2:**
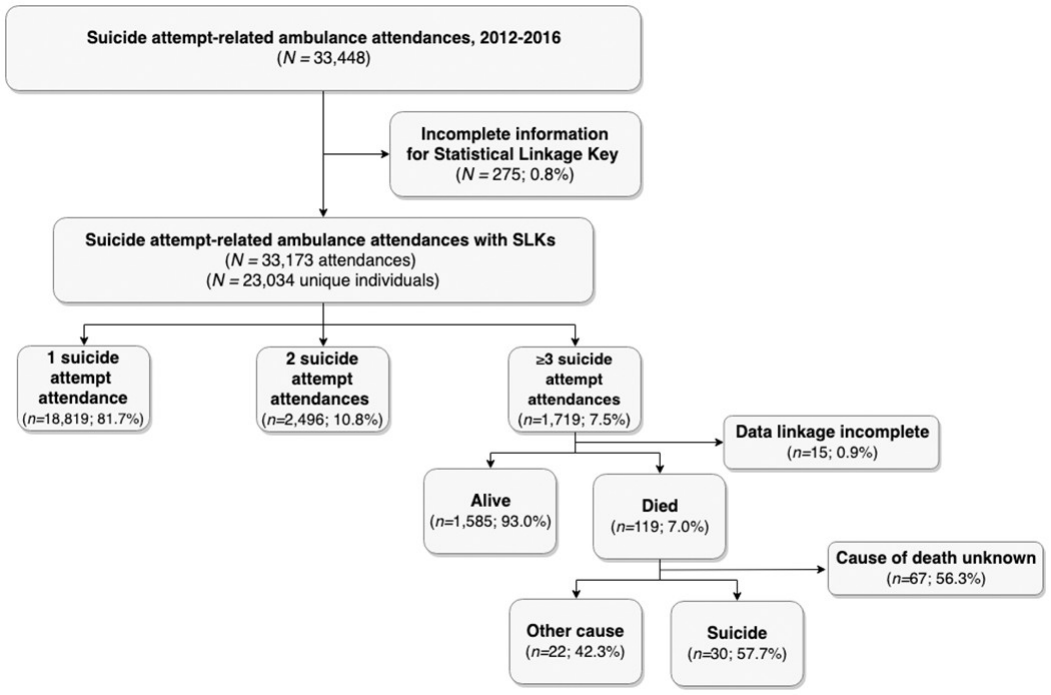
Study flow diagram.

For 275 attendances, insufficient information on patient name, date or birth, or sex was recorded preventing the generation of a statistical linkage key (SLK) to enable the identification of individual patients and to facilitate data linkage. These records were therefore excluded, leaving a total of 33,173 suicide attempt–related attendances over this period (Fig 2).

### Patient characteristics

These 33,173 attendances involved 23,034 individuals (60.0% females, 39.9% males, 0.1% transgender). The number of suicide attempt-related attendances for any one individual ranged between one and 91. Most (*n* = 18,819; 81.7%) made one suicide attempt over this period (Fig 2). As a minimum of three independent observations is required to robustly identify trajectory growth [20], we excluded those with fewer than three suicide attempt–related attendances. All subsequent analyses are therefore based on the 1,719 individuals (68.1% female, 31.8% male, <5 transgender) with three or more suicide attempt-related attendances between 1 January 2012 and 31 December 2016 (Fig 2).

Compared to those with two or fewer suicide attempt–related attendances, those with three or more attendances for attempted suicide were more likely to report a lifetime diagnosis of any anxiety disorder, depression, any psychosis, bipolar disorder, and any personality disorder. They were also more likely to have died from any cause. However, the magnitude of these differences, as measured by Cramér’s V, were negligible to small (Table 1).

**Table 1:** Demographic and clinical characteristics of those with one or two ambulance attendances for suicide attempt (*N* = 21,315) as compared to those with three or more attendances (*N* = 1,719) between 2012 and 2016.

Characteristic	1–2 Attendances (*N* = 21,315)	≥3 Attendances (*N* = 1,719)	OR (95% CI)	Cramér’s V
**Age category**				
*Under 15 years*	630 (3.0)	76 (4.4)	**1**.**52 (1**.**19–1**.**94)**	0.05
*15 to 24 years*	6,104 (28.6)	529 (30.8)	1.08 (0.99–1.23)	
*25 to 44 years*	8,366 (39.2)	688 (40.0)	1.03 (0.93–1.14)	
*45 to 64 years*	4,985 (23.4)	398 (23.1)	0.99 (0.88–1.11)	
*65 years or older*	1,228 (5.8)	27 (1.6)	**0**.**26 (0**.**18–0**.**38)**	
*Missing*	<5	<5	–	–
**Gender**				
*Male*	8,651 (40.6)	546 (31.8)	**0**.**68 (0**.**61–0**.**76)**	0.05
*Female*	12,642 (59.3)	1,171 (68.1)	**1**.**47 (1**.**32–1**.**63)**	
*Indeterminate/Transgender*	22 (0.1)	<5	–	–
*Missing*	0 (0.0)	0 (0.0)	–	–
**Self-reported Lifetime Psychiatric Diagnoses**				
*Substance dependence*	348 (1.6)	19 (1.1)	1.03 (0.65–1.65)	0.13
*Anxiety disorder*	4,811 (22.6)	444 (25.8)	**1**.**19 (1**.**07–1**.**34)**	0.02
*Depression*	12,484 (58.6)	1,058 (61.5)	**1**.**13 (1**.**02–1**.**25)**	0.02
*Psychosis*	1,184 (5.5)	168 (9.8)	**1**.**84 (1**.**55–2**.**18)**	0.05
*Bipolar disorder*	1,481 (6.9)	194 (11.3)	**1**.**70 (1**.**45–2**.**00)**	0.04
*Other Axis 1 disorder*	649 (3.0)	59 (3.4)	1.13 (0.86–1.48)	0.01
*Personality disorder*	884 (4.1)	218 (12.7)	**3**.**36 (2**.**87–3**.**92)**	0.10
**All-cause mortality**				
*Yes*	809 (3.8)	87 (5.1)	**1**.**35 (1**.**08–1**.**69)**	0.02
*No*	20,480 (96.1)	1,630 (94·.8)	0.76 (0.61–0.96)	
*Missing*	26 (0.1)	<5	–	–

**NOTE**. CI: Confidence Interval; OR: Odds Ratio. Boldface indicates covariates significant at the nominal *p*<0.05 level or lower.

### Latent trajectory analyses

Two distinct trajectories provided optimal fit. This model was selected because, compared to a model with one group, it yielded an optimised BIC. Models with more than two groups, on the other hand, were unstable and did not identify additional groups of individuals that were likely to be clinically distinct from those identified by the two group model.

**Fig 3:**
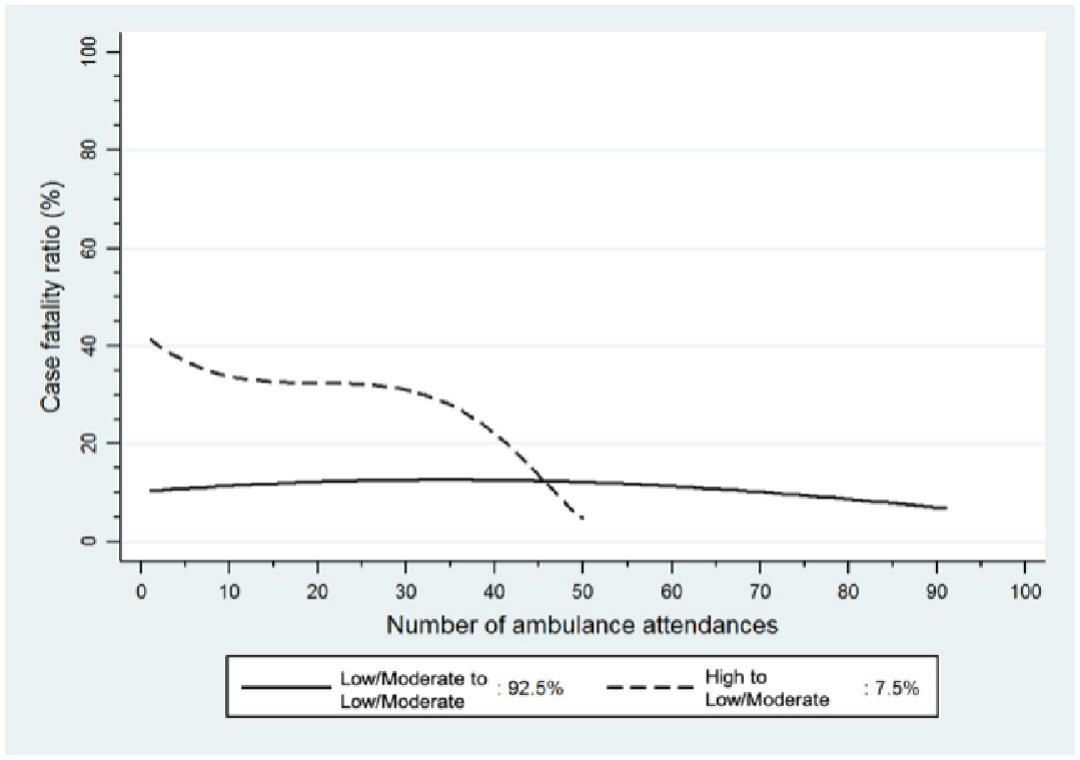
Case fatality ratios of methods used over successive episodes for those with three or more suicide attempt-related ambulance attendances in Victoria, 2012 to 2016 (N = 1,719).

The first trajectory group (Low/Moderate to Low/Moderate Lethality), comprising the majority of those with three of more suicide attempt–related attendances over this period (*n* = 1,600; 92.5%), consisted of those who consistently used methods associated with low to moderate CFRs, such as intentional drug overdose (IDO), poisoning by other substances, contact with sharp objects, and other methods over the course of the observation period. The second group (High to Low/Moderate Lethality), comprising 7.5% of the cohort (*n* = 119), consisted of those who started out using methods associated with higher CFRs, such as hanging, strangulation, and suffocation, use of firearms or explosives, drowning and submersion, and falls from a height but who switched to methods characterised by successively lower CFRs over successive episodes (Fig 3).

Following multivariate adjustment, self–reported depression was associated with decreases in suicide attempt lethality, whilst self–reported psychosis or any other Axis I psychiatric disorder were associated with increases in suicide attempt lethality for both trajectory groups. For those in the High to Low/Moderate Lethality group specifically, self–reported anxiety or any personality disorder were both associated with decreases in suicide attempt lethality. Alcohol intoxication was associated with increases in suicide attempt lethality, but only for those in the Low/Moderate to Low/Moderate Lethality group, whilst self-reported alcohol or drug dependence was also associated with increases in suicide attempt lethality, but only for those in the High to Low/Moderate Lethality group (Table 2).

**Table 2:** Influence of time varying covariates on trajectory membership for those with three or more suicide attempt–related ambulance attendances in Victoria, 2012 to 2016 (*N* = 1,719).

	Univariate Model						Multivariate Adjusted Model					
	Low/Moderate to Low/Moderate Lethality			High to Low/Moderate Lethality			Low/Moderate to Low/Moderate Lethality			High to Low/Moderate Lethality		
	β	*SE*	*p*	β	*SE*	*p*	β	*SE*	*p*	β	*SE*	*p*
**Intercept**	5.33	0.29	<0.001	38.89	1.48	<0.001	7.02	0.49	<0.001	47.29	2.14	<0.001
**Time varying covariates**												
Alcohol intoxication	5.71	0.38	<0.001	2·.27	1.58	0.150	5.23	0.37	<0.001	–3.00	1.48	0.052
Illicit drug use	1.32	0.51	0.010	4.44	3.44	0.196	0.69	0.52	0.187	1.38	2.67	0.606
Substance dependence	1.57	0.48	0.001	**13.73**	**2.70**	**<0.001**	0.09	0.47	0.851	**4.81**	**1.82**	**0.008**
Anxiety disorder	–0.57	0.36	0.115	**–6.06**	**1.54**	**0.001**	–0.17	0.37	0.642	–**2.96**	**1.36**	**0.030**
Depression	–1.63	0.01	0.002	**–15.09**	**3.37**	**<0.001**	–1.88	0.53	<0.001	–**9.02**	**2.38**	**<0.001**
Psychosis	0.52	0.42	0.214	**13.39**	**2.41**	**<0.001**	1.25	0.43	0.004	**3.98**	**1.62**	**0.014**
Bipolar disorder	–0.98	0.42	0.021	**–13.06**	**1.80**	**<0.001**	–0.82	0.43	0.054	–0.94	1.53	0.538
Other Axis I disorder	1.54	0.50	0.002	**9.25**	**1.81**	**<0.001**	1.25	0.49	0.011	**10.38**	**1.58**	**<0.001**
Personality disorder	–0.94	0.41	0.023	**–16.02**	**1.76**	**<0.001**	–0.29	0.41	0.468	–**16.64**	**1.65**	**<0.001**

**NOTE**. β: coefficient; *SE*: standard error; *p*: probability value. Boldface indicates covariates significant at the nominal *p*<0.05 level or lower following multivariate adjustment.

### Suicide attempt repetition

Of the 1,719 cohort members, around one-quarter (*n* = 429, 24.9%) had a further suicide attempt-related ambulance attendance over the follow–up period. As a similar proportion of those assigned to the Low/Moderate to Low/Moderate Lethality group had at least one further ambulance attendance for attempted suicide over this period as those assigned to the High to Low/Moderate Lethality group (24.9% *vs*. 26.0% respectively), there was no significant difference between groups in the hazards of making a subsequent suicide attempt (Hazards Ratio [HR] = 1.41, 95% CI 0.76 to 2.59, *z* = 1.10, *p* = 0.271).

### Suicide mortality

Data linkage was possible for 1,704 (99.1%) members of the cohort, of whom 119 (7.0%) died from any cause over a median follow-up period of 35.3 months (IQR: 21·9 to 54·0 months). For just over half (56·3%), information on cause of death remained open at 31 December 2017 (i.e., end of the follow–up period). As a consequence, these individuals were excluded, leaving 52 cohort members with complete information on cause of death of whom over half (57.7%) had died by suicide (Fig 2). The sub–hazards of dying by suicide appeared to be higher for those assigned to the High to Low/Moderate Lethality group as compared to the Low/Moderate to Low/Moderate Lethality group (Sub–Hazards Ratio [SHR] = 2.82, 95% CI 1.16 to 6.86, *z* = 2.29, *p* = 0.022).

### All–cause mortality

A slightly greater proportion of those assigned to the High to Low/Moderate Lethality group died from any cause during the follow-up period (8.4% *vs*. 6.9% respectively), however, there was no significant difference between trajectory groups in the hazards of all–cause mortality (HR = 1.21, 95% CI 0.63 to 2.32, *z* = 0.59, *p* = 0.555).

## Discussion

In a state-wide cohort of all Victorians attended by ambulance services following a suicide attempt between 1 January 2012 and 31 December 2016, we found the majority (81.7%) made one suicide attempt. One–in–ten (10.8%) made two suicide attempts, and 7.5% made multiple suicide attempts. Amongst those who made multiple suicide attempts, we identified two distinct trajectories in suicide attempt method lethality: one characterised by the use of methods typically associated with lower CFRs, such as IDO and self-cutting (Low/Moderate to Low/Moderate Lethality group), and a second associated with a gradual decrease in method lethality (High to Low/Moderate Lethality group).

A number of clinical factors differentially influenced suicide method lethality between these two groups. Acute alcohol intoxication was associated with increased lethality in the Low/Moderate to Low/Moderate Lethality group. A history of substance dependence (but not necessarily acute intoxication), on the other hand, was associated with increased attempt lethality in the High to Low/Moderate Lethality group in this study. Recent work has found that acute alcohol intoxication may be associated with the use of higher lethality methods, whilst chronic alcohol use in the absence of acute intoxication may be associated with the use of lower lethality methods in suicide decedents [23], and in those who have attempted suicide [24]. However, these studies have rarely considered the impact of changes in these associations over time.

With regards to psychiatric symptomatology, we found that depression was associated with decreased suicide attempt lethality for those assigned to the High to Low/Moderate Lethality group. Although recent work has found that variability in depressive symptoms over time may be a significant marker for incident suicide attempt risk [25], associations were significant only for those with high stable depressive illness courses. Psychosis symptoms were also associated with increased suicide attempt lethality in those assigned to the High to Low/Moderate Lethality group in this study. Whilst we were unable to assess the relative contribution of fluctuations in positive versus negative symptomatology, previous work has suggested that certain positive symptoms (e.g., command hallucinations, threat control override symptoms), as well as levels of symptom-related distress, may be associated with self-harm [26]. Levels of insight might also play a role [27]. However, as so few studies have investigated associations between psychiatric illness trajectories, including symptom severity, with suicide attempt risk, the relative contribution of state versus trait psychiatric symptomatology remains poorly understood at present [28]. Future work in this area could capitalise on the potential offered by ecological momentary assessment (EMA) to assist in the identification of the relative impact of daily symptom fluctuations on self-harm risk, as well as any associations with self-harm method, over time [29].

Although male gender has also been associated with increased use of methods associated with greater potential lethality, we did not find any association between male gender and suicide attempt lethality in either trajectory in this study. However, both trajectory groups predominately comprised females (68.9% and. 57.1% female respectively). Given that males are more likely than females to use methods associated with greater potential lethality, and consequently are more likely to die on their first attempt [30], it may be that males were less likely to be included in our analysis cohort. Consistent with this, when comparing the proportion of males in the analysis cohort with that of the overall sample, we did find that our analysis cohort was less likely to include males (31.8% *vs*. 39.9% respectively).

We also found some evidence of differences between these two groups in terms of the hazards of engaging in further suicidal behaviour. Those assigned to the High to Low/Moderate Lethality group may be at greater risk of dying by suicide. However, whilst the hazards of having a further ambulance attendance for attempted suicide were 41% higher in those assigned to the High to Low/Moderate Lethality group as compared to the Low/Moderate to Low/Moderate Lethality group, this difference did not reach conventional levels of significance. Additionally, whilst those assigned to the High to Low/Moderate Lethality group were 21% more likely to die from any cause, there were also no significant differences in the hazards of all–cause mortality between these two groups.

### Strengths

Previous studies have modelled hazards until the first repeat episode. However, as any one individual can engage in multiple repeat episodes over the course of the follow–up period, these models are inefficient as data on later episodes are discarded [31]. In contrast, our models were extended to account for multiple repeat events. Models were also adjusted for autocorrelation between repeated events in order to protect against inflation of the type I error rate [31]. Our models for suicide death were also extended to account for competing risks [32].

We also believe our cohort is representative. Ambulance Victoria is the sole emergency medical service provider in the state of Victoria, Australia [33]. Ambulance coverage is provided free of charge to all persons receiving government entitlements, including those on disability and low-income pensions [34]. For those not receiving government entitlements, an opt–in membership subscription is available (at the nominal cost of 47 Australian Dollars per annum at the time of writing) [34]. Many private health insurance policies also include ambulance membership. However, if an individual is not covered, an ambulance will still be dispatched as Ambulance Victoria does not charge for services rendered at the point of use. Additionally, one–quarter (25.6%) of Australia’s population lived in the state of Victoria as at 31 December 2016 (i.e., the end of the observation period) [18].

### Limitations

Our data–capture mechanism excluded suicide attempts occurring in the community for which an ambulance did not attend. There may be important differences in the characteristics of those who make suicide attempts in the community as compared to those who come to the attention of clinical services. Recent work has found that self–injury may be more common than IDO in both males and females in the community [35]. In contrast, IDO is significantly more common than some forms of self-injury (e.g., self–cutting), particularly in females, in hospital–presenting populations.

Additionally, although the majority of our cohort (98.6%) were transported to the emergency department, and further, the majority of emergency department presentations for non–fatal self–harm in Victoria involve ambulance transportation [36], there may nonetheless be important differences between those who are attended by ambulance as compared to those who self-present to the emergency department; although a recent data linkage study found the risks of both all–cause and suicide mortality may be similar [37].

Second, the observation period may have been affected by both left and right censoring, common to all studies in this area. The start of our observation period represents an arbitrary point and it could well be that some members of the cohort will have attempted suicide in previous years [38]. Similarly, we were only able to follow cohort members until the date of their death or 31 December 2017 (whichever came first). Therefore, for those 1,585 cohort members alive at 31 December 2017, data are right censored as these individuals may well continue to engage in further suicide attempts beyond the end of the follow-up period. Therefore, for some, the patterns we observe in this study may reflect only the latest within a longer series.

We included only those with three or more attendances for attempted suicide during the observation period as latent trajectories identified from fewer than three independent observations are likely to be unstable [20], and may capitalise on chance associations. It is also important to note that our aim was to determine whether there is evidence of gradual changes in method lethality in those engaging in successive repeat episodes of non–fatal self–harm which we defined, consistent with the international literature, as those engaging in three or more episodes [6]. Additionally, whilst international data would suggest that the majority of suicide deaths are first attempts [30], it is not presently known what proportion of first attempts are fatal in Australia due to the lack of comprehensive surveillance systems for non–fatal self-harm in Australia at present [39], and the difficulties in conducting data linkage at the national level [40, 41].

Finally, we relied on mortality data to determine cause of death. An estimated three–quarters of suicide inquest cases in Australia remain open at one year, and 37.5% at two years [42], information on cause of death was unavailable for just over half (56.3%) of the cohort. Whilst we considered undertaking sensitivity analyses to account for this, these analyses would have lacked sufficient statistical power given the model parameters observed. Further work using longer lag times is therefore currently being undertaken to address this limitation.

### Clinical implications

Encouragingly, we found no evidence for method escalation across successive attempts in this study [43], even amongst those who initially used methods associated with higher potential lethality; predominantly hanging, strangulation, and suffocation. However, those assigned to the High to Low/Medium trajectory group did appear to be at increased risk of suicide. Although we were unable to investigate the specific association between trajectory group membership and specific categories of suicide method as such models would have been under–powered, nevertheless, our results do point to the importance of attending to the overall sequence of methods used, including any previous use of methods associated with high potential lethality. Routine assessment of access to potentially lethal means is also strongly encouraged for all persons presenting to clinical services irrespective of the method used at the index presentation [44].

Presently, we cannot determine the extent to which the decline in suicide attempt method lethality observed in this study, particularly for those assigned to the High to Low/Moderate Lethality group, may result from any beneficial effect of treatment. Future work using agent-based analyses to track individuals’ use of psychiatric and ancillary treatment services over time may be required to determine the effect of receiving treatment on these trajectories, and furthermore, whether existing treatments for self-harm are differentially effective for those following the Low/Moderate to Low/Moderate Lethality group trajectory as compared to the High to Low/Moderate Lethality group.

## Conclusions

Our study is the first to model individual variation in patterns of suicide attempt lethality. The majority (92.5%) of those making multiple suicide attempts consistently used methods associated with lower potential lethality, such as IDO and self–cutting. Encouragingly, even in those using methods associated with higher potential lethality, such as attempted hanging, suicide attempt method lethality decreased across successive attempts. However, whilst there were no significant differences between groups in the hazards of reattempting suicide or all–cause mortality, those assigned to the High to Low/Moderate Lethality trajectory group may be at greater risk of suicide, highlighting the importance of assessing for any previous use of methods associated with higher potential lethality in all patients presenting to clinical services irrespective of the method used at the index episode.
